# Different rhizosphere soil microbes are recruited by tomatoes with different fruit color phenotypes

**DOI:** 10.1186/s12866-022-02620-z

**Published:** 2022-08-31

**Authors:** Siyu Chen, Yan Sun, Yufei Wei, Huan Li, Shangdong Yang

**Affiliations:** National Experimental Teaching Demonstration Center of Plant Science, Agricultural College, Guangxi University, Nanning, 530004 Guangxi P.R. China

**Keywords:** Tomato, Bacteria, Fungi, Rhizosphere, Fruit color

## Abstract

**Background:**

To explore and utilize abundant soil microbes and their beneficial functions, the bacterial and fungal compositions in rhizospheres between red- and yellow-fruited tomato varieties were analyzed using high-throughput sequencing technique.

**Result:**

Our results indicated that different soil microbes in rhizospheres of tomatoes were exactly recruited by different color fruit tomatoes. For the reasons as not only soil bacterial community, but also soil fungal compositions were all different between red and yellow fruit tomatoes. For example, *Nocardioides*, *norank_f_norank_o_Vicinamibacterales*, *norank_f_norank_o_norank_c_KD4-96*, *norank_f_Birii41*, *norank_f_norank_o_S085* and *Bradyrhizobium* were the specific dominant soil bacterial genera, and *Lecythophora*, *Derxomyces* and *unclassified_f_Pyronemataceae* were the dominant soil fungal genera in the rhizospheres of red tomato varieties. By contrast, *unclassified_f__Micromonsporaceae*, *Acidipila*, *Roseisolibacter*, Gaiella and *norank_f_Xanthobacteraceae* were the unique dominant soil bacterial genera in the rhizospheres of yellow tomato varieties. And *unclassified_o__Onygenales*, *Trichocladium*, *unclassified_c__Sordariomycetes*, *Pseudogymnoascus*, *Acremonium*, *Oidiodendron*, *Phialemonium*, *Penicillium*, *Phialosimplex* were the unique dominant soil fungal genera in rhizospheres of yellow tomato varieties. Moreover, a higher abundance of specific soil bacterial and fungal genera in the rhizosphere was found in rhizospheres of the yellow than those of the red tomato varieties.

**Conclusion:**

Soil bacterial and fungal compositions in rhizospheres between red- and yellow-fruited tomato varieties were found significantly different which growing in the same environment under the identical managements. It suggested that different soil microbes in rhizospheres exactly were recruited by different phenotypes tomato varieties related to fruit color formation.

## Background

Tomato (*Lycopersicon esculentum* Mill.) fruits may have a wide range of colors, such as red, yellow, purple and orange [1]. As is well known, fruit color is one of the most important commercial qualities of tomatoes because it is used as one of the important factors in the evaluation of the nutritional quality of tomatoes [2].

The range of tomato fruit colors depends mainly on the composition and proportion of pigments contained in the fruit. Carotenoids and anthocyanins are the main pigments in tomato fruit [3]. Previous studies have shown that abscisic acid promotes carotenoid biosynthesis in tomatoes [4]; ethylene also showed a function in promoting carotenoid accumulation in tomatoes [5]. Moreover, auxin could also promote lycopene synthesis and enhance red colors in tomato fruits [6]. Moreover, previous studies have shown that erythromycin influences the synthesis of anthocyanins [7]. Strigolactone has a positive effect on anthocyanin accumulation [8], and jasmonic acid and abscisic acid can synergize with sugars in the anthocyanin synthesis pathway [9].

It is well known that microbes play an important role in soil, particularly in the rhizospheres of plants, and have interdependent relationships with plants [10, 11]. Among them, soil bacteria can produce many plant growth regulators [12, 13], such as auxin [14], gibberellin [15], cytokinin [16], ethylene [17] and abscisic acid [18]. All of the above phytohormones, which are derived from soil microbes, can be taken up by plants and affect plant growth either directly or indirectly by influencing the rhizosphere environment [19, 20]. In addition, fungi also closely interact with plants, producing various phytohormones affecting the endogenous hormone levels of the plants [21], such as auxin [22], abscisic acid [23], jasmonic acid and salicylic acid [24], ethylene [25, 26], erythromycin [27], and cytokinin [28].

Although fruit color formation has been linked to microbes, the kinds of microbes in soil that are related to carotenoid or anthocyanin biosynthesis in tomato fruits are still unclear. Therefore, to elucidate what kinds of soil microbes are related to carotenoid or anthocyanin biosynthesis, the compositions of the soil microbial community in the rhizospheres of yellow and red fruit tomatoes were analyzed.

## Results

### Soil bacterial diversity and richness in rhizospheres of tomato varieties with different fruit colors

The Ace and Chao1 indices, which describe soil bacterial richness, were not significant differences in rhizospheres of yellow and red tomato varieties and background. Moreover, the soil bacterial diversity, which describes with the Shannon and Simpson indices, were not significant differences among the background, yellow and red tomato varieties too (Table 1).

**Table 1:** Richness and diversity of soil bacteria in rhizospheres of tomato varieties with different fruit colors; Data in the table are means ± SDs. Values followed by different lowercase letters indicate significant differences between soil bacteria in varieties with different fruit colors (*p* < 0.05)

Sample	Shannon index	Simpson index	Ace index	Chao1 index	Coverage
yellow tomato varieties (Y)	5.64 ± 0.55a	0.01 ± 0.0075a	2219.45 ± 576.08a	2174.51 ± 577.15a	0.98
red tomato varieties (R)	6.06 ± 0.27a	0.01 ± 0.0025a	2708.90 ± 440.74a	2647.23 ± 335.59a	0.98
background (CK)	5.94 ± 0.54a	0.01 ± 0.0064a	2416.75 ± 659.66a	2405.37 ± 591.13a	0.98

### Compositions of soil bacterial communities in rhizospheres of tomato varieties with different fruit colors

**Fig. 1:**
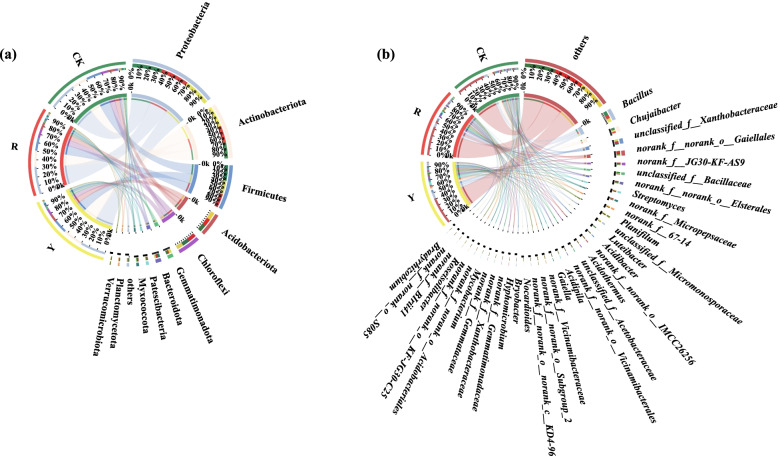
Proportion of dominant rhizosphere soil bacteria for yellow- (Y) and red-fruited tomatoes (R) and background soil (CK). **a**: Soil bacteria at the phylum classification level; (**b**): Soil bacteria at the genus classification level

As shown in Fig. 1a, the numbers of dominant soil bacterial phyla (i.e., relative abundances were greater than 1%) among the background, yellow and red tomato varieties were 11, 10, 12, respectively.

Firstly, Proteobacteria (33.43%), Actinobacteriota (18.63%), Firmicutes (17.85%), Chloroflexi (8.73%), Acidobacteriota (8.47%), Bacteroidota (3.38%), Nitrospirae (2.89%), Planctomycetota (1.68%), Myxococcota (1.55%), Patescibacteria (1.47%), Verrucomicrobia (1.29%) and others (1.87%) were the dominant soil bacterial phyla of CK.

In addition, Proteobacteria (30.39%), Actinobacteriota (24.27%), Firmicutes (16.31%), Acidobacteriota (9.40%), Chloroflexi (7.84%), Gemmatimonadota (3.74%), Bacteroidota (2.34%), Myxococcota (1.38%), Planctomycetota (1.32%) and others (1.30%) were the dominant soil bacterial phyla in the rhizosphere of yellow tomato varieties.

In contrast, Proteobacteria (32.59%), Actinobacteriota (19.20%), Firmicutes (15.74%), Chloroflexi (9.83%), Acidobacteriota (9.04%), Gemmatimonadota (2.78%), Bacteroidota (2.44%), Patescibacteria (2.24%), Myxococcota (2.03%), and others (1.64%) were the dominant soil bacterial phyla in the rhizospheres of red tomato varieties.

Among them, Gemmatimonadota, accumulated as the dominant soil bacterial phyla in the rhizospheres of the yellow or red tomato varieties which compared with CK. Patescibacteria and Verrucomicrobia were the unique dominant soil bacterial phyla in the rhizospheres of red tomato varieties. Although the soil bacterial compositions at phylum level in the rhizospheres of the yellow or red tomato varieties were similar to those of CK, but their proportions were different. The result suggested that the soil bacterial compositions at phylum level in rhizospheres of tomatoes strongly followed the soil bacterial compositions in background (CK), but also it indicated that the proportions of soil dominant bacteria in rhizospheres of tomatoes at phylum level could be altered by planting with different tomato varieties. Furthermore, dominant soil bacterial genera (i.e., those with relative abundances were greater than 1%) among the CK, yellow- (Y) and red-fruited tomato varieties (R) numbered 24, 26 and 27, respectively (Fig. 1b).

First, *Bacillus* (6.57%), *Chujaibacter* (4.38%), *Luteibacter* (3.73%), *norank_f_norank_o_Gaiellales* (3.15%), *unclassified_f__Bacillaceae* (2.59%), *unclassified_f__Xanthobacteraceae* (2.38%), *norank_f_norank_o_Elsterales* (1.98%), *norank_o_Vicinamibacterae* (1.85%), *norank_f_JG30-KF-AS9* (1.81%), *Planifilum* (1.59%), *Mycobacterium* (1.47%), *norank_f_norank_o_KF-JG30-C25* (1.39%), *norank_f_Gemmatiaceae* (1.38%), *norank_f_norank_o_Vicinamibacterales* (1.37%), *norank_f_norank_o_norank_c_KD4-96 *(1.37%), *norank_f_norank_o_Vicinamibacteraceae* (1.37%), *unclassified_f__Acetobacteraceae* (1.37%), *Acidibacter* (1.35%), *norank_f_norank_o_IMCC26256* (1.33%), *Streptomyces* (1.31%), *norank_f_Micropepsaceae* (1.23%), *Nocardioides* (1.04%), *Gaiella* (1.02%), *1.02* and others (45.05%) were the dominant bacterial genera in CK.

Second, *Bacillus* (6.02%), *unclassified_f__Micromonsporaceae* (3.87%), *unclassified_f__Xanthobacteraceae* (3.79%), *Chujaibacter* (3.03%), *Streptomyces* (2.80%), *norank_f_67-14* (2.71%), *norank_f_norank_o_Gaiellales* (2.49%), *Acidipila* (2.24%), *unclassified_f__Bacillaceae* (2.02%), *norank_f_norank_o_Elsterales* (1.90%), *norank_f_Micropepsaceae* (1.90%), *Acidothermus* (1.82%), *norank_f_JG30-KF-AS9* (1.80%), *Planifilum* (1.75%), *Roseisolibacter* (1.60%), *Gaiella* (1.46%), *norank_f_Xanthobacteraceae* (1.30%), *Hyphomicrobium* (1.28%), *norank_f_norank_o_IMCC26256* (1.25%), *norank_f_Gemmatimonadaceae* (1.23%), *unclassified_f__Acetobacteraceae* (1.20%), *Acidibacter* (1.15%), *Bryobacter* (1.11%), *norank_f_norank_o_Acidobacteriales* (1.08%), *norank_f_norank_o_Subgroup_2* (1.02%) and others (42.02%) were the dominant soil bacterial genera in the rhizospheres of yellow tomato varieties.

In contrast, *Bacillus* (6.32%), *norank_f_norank_o_Gaiellales* (4.01%), *unclassified_f__Xanthobacteraceae* (3.67%), *Chujaibacter* (2.93%), *norank_f_JG30-KF-AS9* (2.72%), *norank_f_norank_o_Elsterales* (2.35%), *norank_f_Micropepsaceae* (2.13%), *Streptomyces* (1.90%), *unclassified_f__Bacillaceae* (1.63%), *Planifilum* (1.52%), *norank_f_67-14* (1.47%), *Acidibacter* (1.43%), *norank_f_norank_o_Subgroup_2* (1.38%), *norank_f_norank_o_IMCC26256* (1.33%), *Nocardioides* (1.30%), *norank_f_norank_o_Vicinamibacterales* (1.22%), *norank_f_Gemmatimonadaceae* (1.19%), *unclassified_f__Acetobacteraceae* (1.17%), *norank_f_norank_o_norank_c_KD4-96* (1.16%), *Bryobacter* (1.13%), *Acidothermus* (1.07%), *Hyphomicrobium* (1.07%), *norank_f_norank_o_Acidobacteriales* (1.04%), *norank_f_Birii41* (1.04%), *norank_f_norank_o_S085* (1.03%), *Bradyrhizobium* (1.01%) and others (44.62%) were the dominant soil bacterial genera in the rhizospheres of red tomato varieties.

Based on the above results, *Nocardioides*, *norank_f_norank_o_Vicinamibacterales*, *norank_f_norank_o_norank_c_KD4-96*, *norank_f_Birii41*, *norank_f_norank_o_S085* and *Bradyrhizobium* were found as the special dominant soil bacterial genera in the rhizospheres of red tomato varieties; By contrast, *unclassified_f__Micromonsporaceae*, *Acidipila*, *Roseisolibacter*, *Gaiella* and *norank_f_Xanthobacteraceae* were the unique dominant soil bacterial genera in the rhizospheres of yellow tomato varieties.

**Fig. 2:**
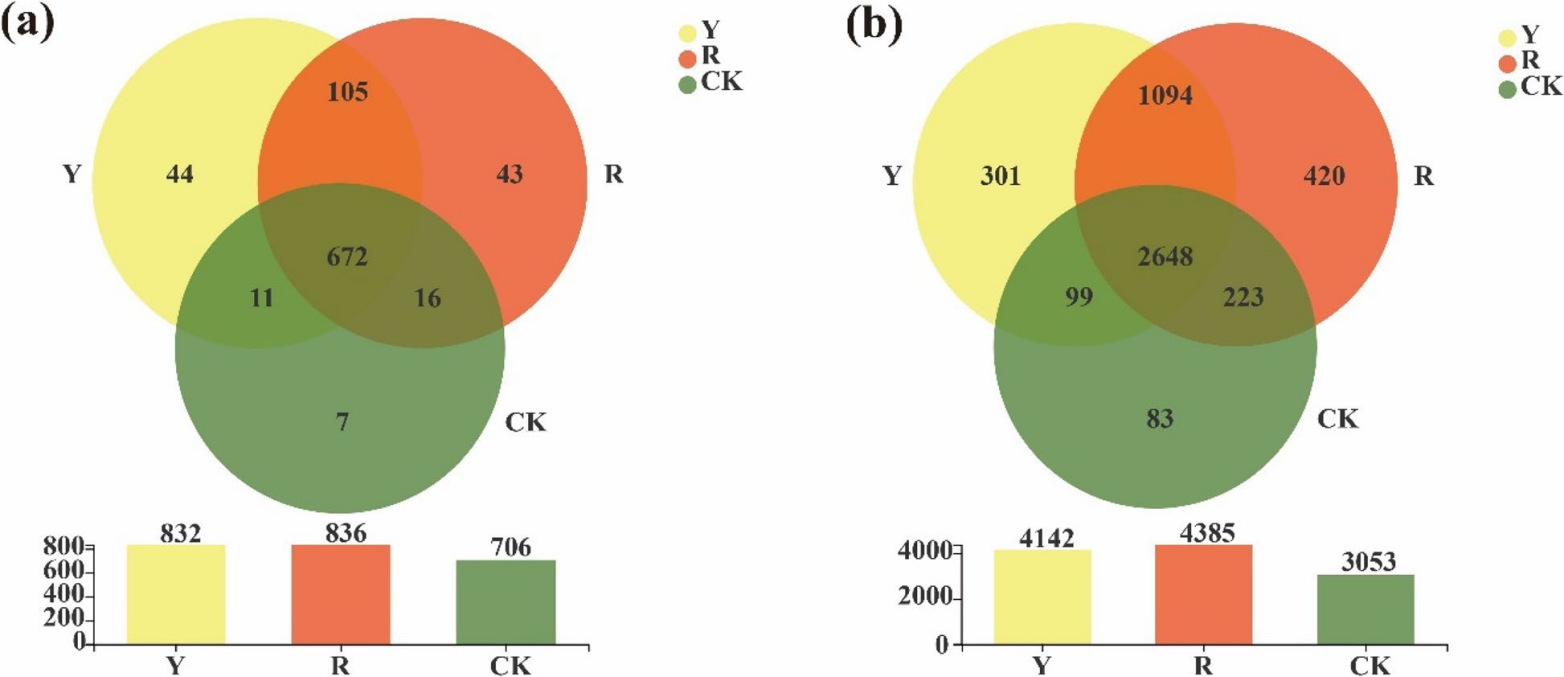
Venn diagram of soil bacteria among CK and the rhizospheres of yellow- (Y) and red-fruited (R) tomato varieties; (**a**): Soil bacteria at the genus level; (**b**): Soil bacteria at the OTU level

Furthermore, the numbers of soil bacteria obtained in the rhizospheres of yellow and red tomato varieties and CK at the genus level were 832, 836, and 706, respectively (Fig. 2a). Moreover, the numbers of unique bacteria at genus level in rhizospheres of yellow and red tomato varieties and CK were 44, 43 and 7, respectively. In addition, the numbers of soil bacteria obtained at the OTU level in rhizospheres of yellow and red tomato varieties and CK were 4,142, 4,385, and 3,053, respectively. Among them, the numbers of unique bacteria at OTU level in rhizospheres of yellow and red tomato varieties and CK were 301, 420 and 83, respectively (Fig. 2b). These results suggested that the soil bacterial community structure in rhizospheres could be significantly shaped by the tomato varieties, and the numbers of bacteria in the rhizosphere of red fruit tomato varieties were higher than those of yellow fruit tomato varieties. It also indicated that the red fruit tomato varieties recruited more complicated bacteria to help them in accomplishing their growth.

**Fig. 3:**
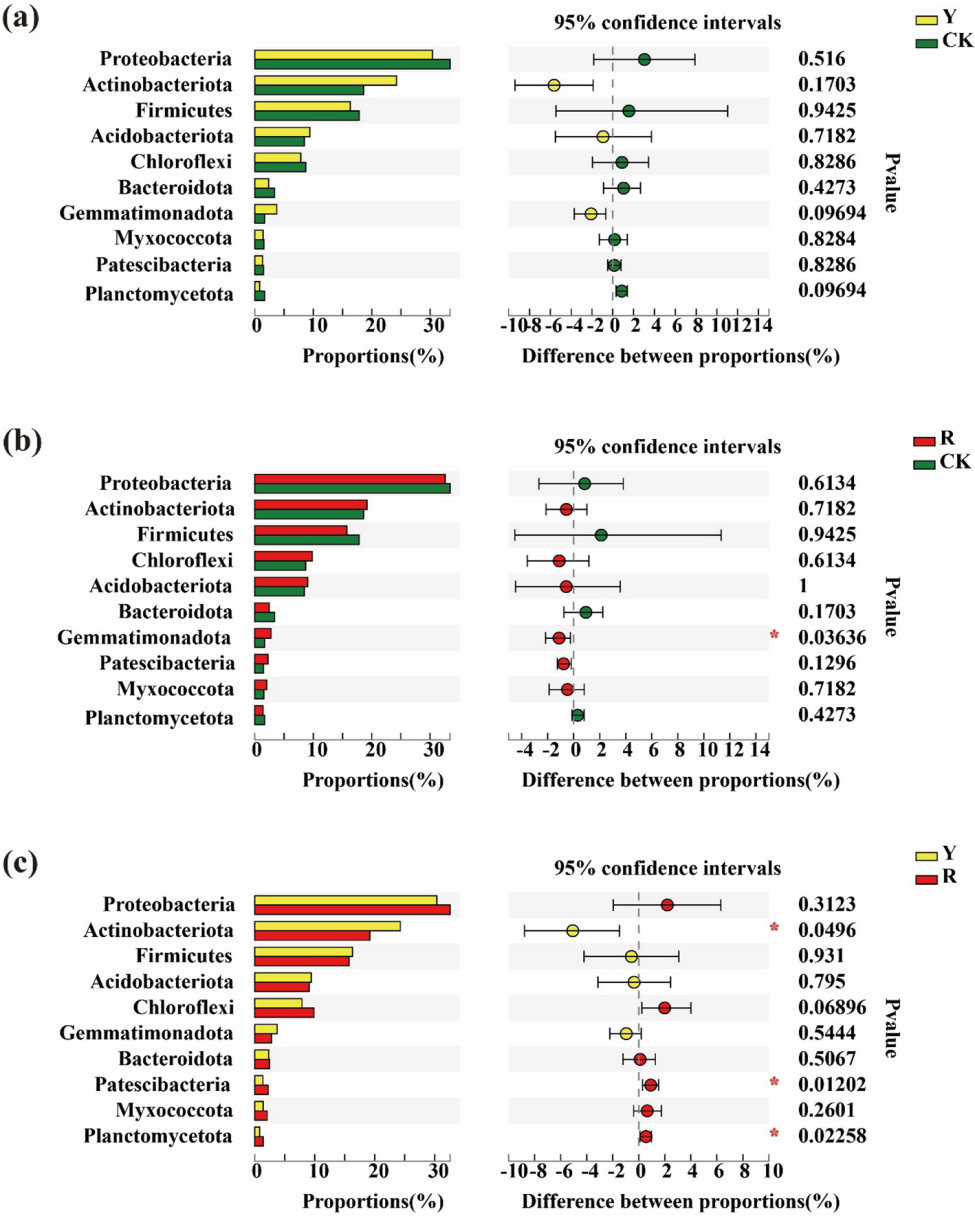
Difference test of dominant soil bacteria in rhizospheres at the phylum among CK and the rhizospheres of yellow- (Y) and red-fruited (R) tomato varieties *0.01 < *p* ≤ 0.05, **0.001 < *p* ≤ 0.01, ***p ≤ 0.001

Furthermore, based on the relative abundance data, significant differences in the 15 top soil bacteria at the phylum level in the rhizospheres of different tomato varieties were analyzed using the Wilcoxon rank-sum test. As shown in Fig. 3, they were no significantly different between yellow tomato varieties and CK (Fig. 3a). However, Gemmatimonadota were significantly different between red tomato varieties and CK (Fig. 3b). Furthermore, Actinobacteriota, Patescibacteria and Planctomycetota were significantly different in rhizospheres between yellow and red tomato varieties (Fig. 3c) (Wilcoxon rank-sum test, *p* < 0.05, *p* < 0.01).

**Fig. 4:**
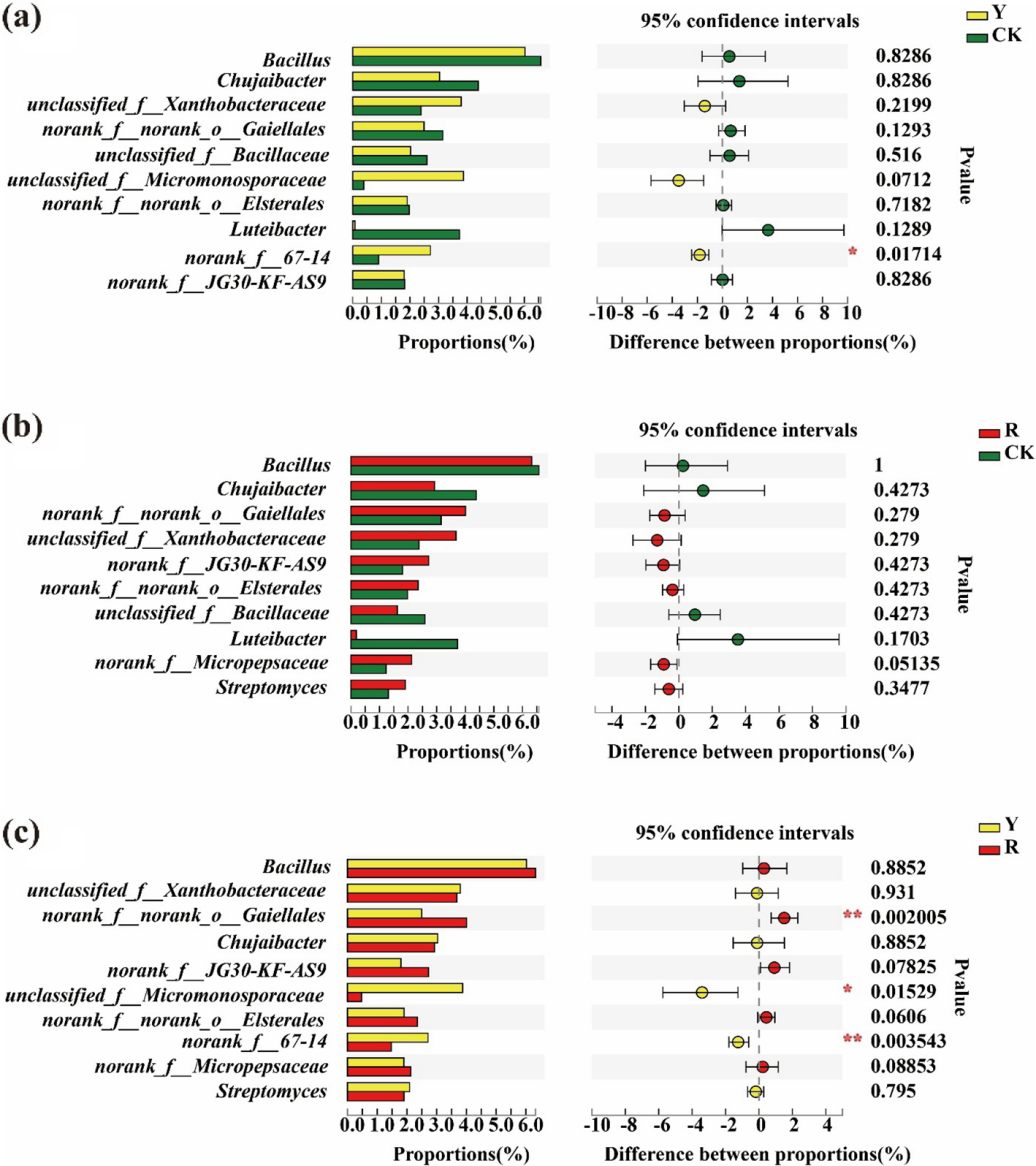
Difference test of dominant soil bacteria in the rhizosphere at the genus level among CK and the rhizospheres of yellow- (Y) and red-fruited (R) tomato varieties; *0.01 < *p* ≤ 0.05, **0.001 < *p* ≤ 0.01, ***p ≤ 0.001

Moreover, significant differences in the 15 top bacteria at the genus level in the rhizospheres of two different tomato varieties were also analyzed. As shown in Fig. 4, *norank_f__67-14* were significantly different between yellow tomato varieties and CK (Fig. 4a). However, they were no significantly different between red tomato varieties and CK (Fig. 4b). But, *norank_f__norank_o__Gaiellales*, *unclassified_f__Micromonosporaceae and norank_f__67-14* were significantly different in the rhizospheres between yellow and red tomato varieties (Fig. 4c) (Wilcoxon rank-sum test, *p* < 0.05, *p* < 0.01).

**Fig. 5:**
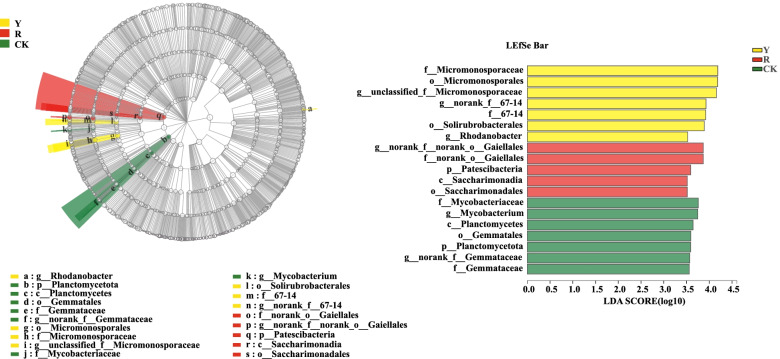
Lefse analysis of significant bacteria among CK and the rhizospheres of yellow- (Y) and red-fruited (R) tomato varieties

A nonparametric factorial Kruskal–Wallis (KW) rank sum test and LEfSe analysis (LDA threshold of 3.5) were carried out to analyze the significant differences and the main contributing biomarker classes between yellow and red tomato varieties. A greater LDA score indicated a greater influence on species abundance under differential effects (Fig. 5).

As shown in Fig. 5, the soil bacterial compositions were found significantly different in rhizospheres of yellow- and red-fruited tomato varieties along with the CK. *Rhodanobacter* (genus), Micromonosporales (from order to genus), Solirubrobacterales (from order to genus) were enriched in the rhizospheres of yellow fruit tomato varieties. In contrast, norank_o__Gaiellales (from family to genus), Patescibacteria (phyla), Saccharimonadia (from class to order) were enriched in rhizospheres of red fruit tomato varieties.

**Fig. 6:**
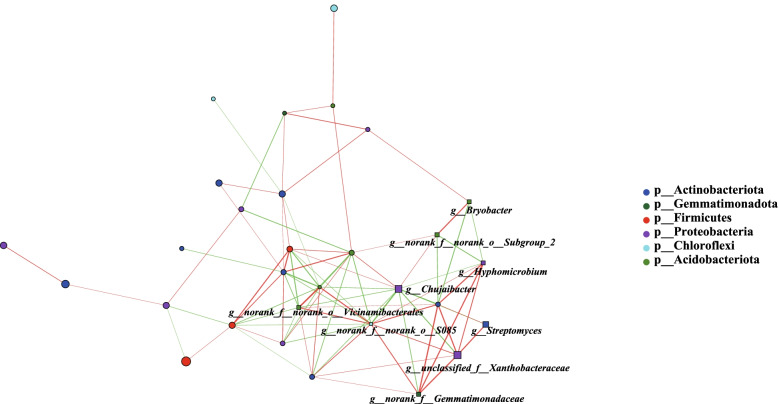
Co-occurrence network analysis of soil bacteria in rhizospheres of different tomato varieties; The red line indicates a positive interaction, the green line indicates a negative interaction, and marked nodes represent significant differences, *p* < 0.05

In addition, the strongest correlation was detected between the genus *norank_f__norank_o__S085* and the other bacteria (Fig. 6). Moreover, most of them were positive connections, such as being positively related to *norank_f__norank_o__Vicinamibacterales*, *norank_f__Gemmatimonadaceae*, *unclassified_f__Xanthobacteraceae*. *Hyphomicrobium* also showed their own connection networks with other bacteria. For example, *norank_f__Gemmatimonadaceae*, *unclassified_f__Xanthobacteraceae* was positively related to *norank_f_Chitinophagaceae*, but *Acidibacter* and *norank_f__norank_o__Subgroup_2*, *Bryobacter* were more negatively associated with *Hyphomicrobium* (Fig. 6).

### Soil fungal diversity and richness in the rhizospheres of tomato varieties with different fruit color

As seen in Table 2, the Shannon and Simpson indices, which describe the soil fungal diversity in rhizospheres between yellow and red tomato varieties, were not significantly different from each other. Furthermore, the Ace and Chao1 indices, which described the soil fungal richness in the rhizosphere of yellow and red tomato varieties, were also not significantly different from each other (Table 2).

**Table 2:** Richness and diversity of soil fungi in rhizospheres of tomato varieties with different fruit colors; Data in the table are means ± SDs. Values followed by different lowercase letters indicate significant differences between soil fungi associated with varieties with different fruit colors (*p* < 0.05)

Sample	Shannon index	Simpson index	Ace index	Chao1 index	Coverage
yellow tomato varieties (Y)	2.53 ± 0.25a	0.16 ± 0.06a	204.83 ± 55.47a	198.25 ± 47.65a	0.99
red tomato varieties (R)	2.32 ± 0.31a	0.18 ± 0.07a	216.94 ± 33.35a	214.13 ± 32.95a	0.99
CK	2.31 ± 0.67a	0.18 ± 0.11a	196.68 ± 29.98a	202.26 ± 39.59a	0.99

### Compositions of soil fungal communities in rhizospheres of tomato varieties with different fruit colors

The numbers of dominant soil fungal phyla (i.e., those with relative abundances greater than 1%) among the background, yellow and red tomato varieties were all 4, respectively.

**Fig. 7:**
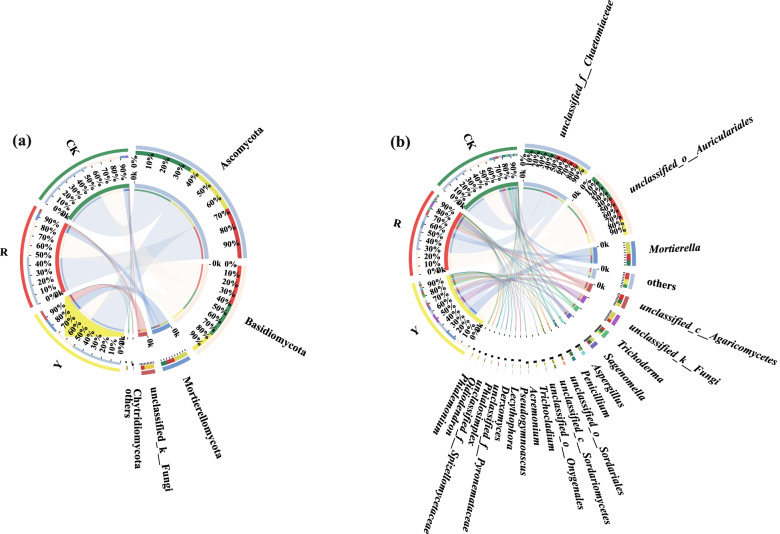
Proportion of dominant rhizosphere fungi in yellow-fruited tomato (Y), red-fruited tomato (R), and background soil (CK), (**a**): Soil fungi at the phylum classification level; (**b**): Soil fungi at the genus classification level

First, Ascomycota (56.26%), Basidiomycota (35.36%), Mortierellomycota (5.98%), Chytridiomycota and (1.81%) were the dominant soil fungal phyla in CK. Second, Ascomycota (52.04%), Basidiomycota (22.44%), Mortierellomycota (14.46%) and unclassified_k_Fungi (10.78%) were the dominant soil fungal phyla in rhizospheres of yellow tomato varieties. By contrast, Ascomycota (49.63%), Basidiomycota (38.54%), Mortierellomycota (8.26%) and unclassified_k_Fungi (3.36%) were the dominant soil fungal phyla in rhizospheres of red tomato varieties (Fig. 7a).

Furthermore, the d soil ominant fungal genera (i.e., those with relative abundances greater than 1%) among the CK, yellow and red tomato varieties were 11, 19, and 13, respectively.

*unclassified_f_Chaetomiaceae* (36.26%), *unclassified_o__Auriculariales* (30.13.%), *Mortierella* (5.98%), *unclassified_c__Agaricomycetes* (5.16.%), *Penicillium* (3.99%), *Trichoderma* (3.55.%), *Sagenomella* (3.03%), *Aspergillus* (2.80%), *unclassified_f__Spizellomycetaceae* (1.80%), *unclassified_o__Sordariales* (1.53%), and others (4.00%) were the soil dominant fungal genera in CK.

In addition, *unclassified_o__Auriculariales* (17.95%), *Mortierella* (14.46%), *unclassified_f__Chaetomiaceae* (14.07%), *unclassified_k__Fungi* (10.78%), *Sagenomella* (4.97%), *unclassified_c__Agaricomycetes* (4.09%), *unclassified_o__Onygenales* (3.46%), *Trichocladium* (3.41%), *Trichoderma* (3.21%), *unclassified_c__Sordariomycetes* (2.70%), *Pseudogymnoascus* (2.57%), *Acremonium* (2.41%), *Aspergillus* (1.98%), *Oidiodendron* (1.50%), *Phialemonium* (1.49%), *Penicillium* (1.32%), *unclassified_o__Sordariales* (1.21%), *Phialosimplex* (1.20%), and others (7.06%) were the soil dominant fungal genera in rhizosphere of yellow tomato varieties. By contrast, *unclassified_o__Auriculariales* (29.12%), *unclassified_f__Chaetomiaceae* (26.89%), *Mortierella* (8.25%), *unclassified_c__Agaricomycetes* (6.67%), *Trichoderma* (4.14%), *unclassified_k__Fungi* (3.36%), *Lecythophora* (2.46%), *Sagenomella* (2.42%), *Derxomyces* (2.2%), *unclassified_f_Pyronemataceae* (1.91%), *unclassified_o__Sordariales* (1.72%), *Aspergillus* (1.12%) and others (7.85%) were the soil dominant fungal genera in rhizosphere of red tomato varieties.

Furthermore, *Lecythophora*, *Derxomyces* and *unclassified_f_Pyronemataceae* were the unique soil dominant fungal genera in the rhizospheres of red tomato varieties. However, *unclassified_o__Onygenales*, *Trichocladium*, *unclassified_c__Sordariomycetes*, *Pseudogymnoascus*, *Acremonium*, *Oidiodendron*, *Phialemonium*, *Penicillium*, *Phialosimplex* were the unique soil dominant fungal genera in the rhizosphere of yellow tomato varieties (Fig. 7b).

**Fig. 8:**
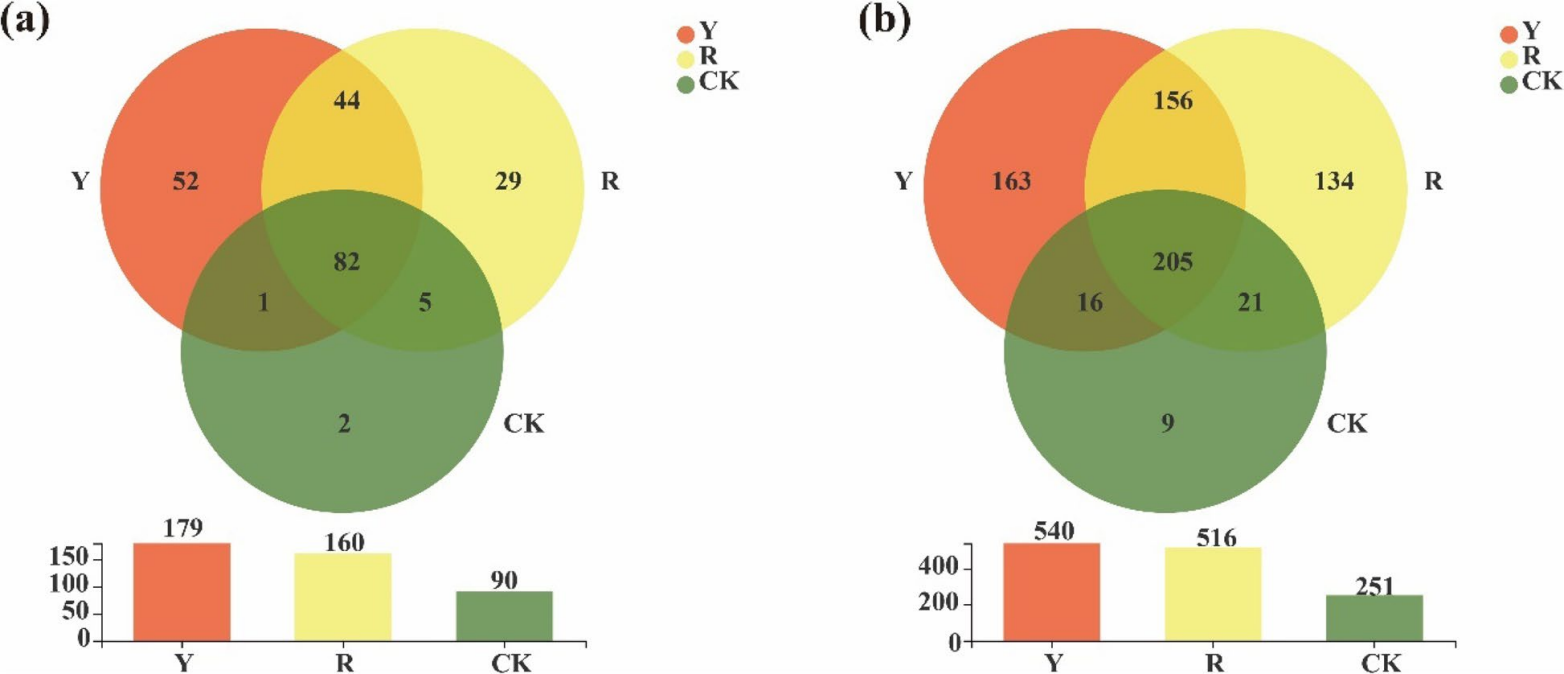
Venn diagram of soil fungi among CK and rhizospheres of yellow-(Y) and red (R)-fruited tomato varieties, (**a**): Soil fungi at the genus level;(**b**): Soil fungi at the OTU level

As seen at Fig. 8, the numbers of soil fungi obtained at the genus level in the rhizospheres of yellow- and red-fruited tomato varieties and CK were 179, 160 and 90, respectively. Moreover, the numbers of unique fungi in the rhizospheres of yellow and red tomato varieties and CK at the genus level were 52, 29 and 2, respectively (Fig. 8a). Similarly, the numbers of soil fungi obtained at the OTU level in the rhizospheres of yellow and red tomato varieties and CK were 540, 516 and 251, respectively. The numbers of unique fungi in the rhizospheres of yellow and red tomato varieties and CK at the OTU level were 163, 134 and 9, respectively (Fig. 8b).

All above results suggested that different soil microorganisms enriched in rhizospheres between red and yellow tomato varieties. In comparison with red tomato varieties, higher abundant of bacteria and fungi were recruited in rhizospheres of yellow tomato varieties.

In addition, significant differences among CK and rhizospheres of yellow and red tomato varieties and the main contributing biomarker classes were also examined by LEfSe analysis (LDA threshold of 3.5).

**Fig. 9:**
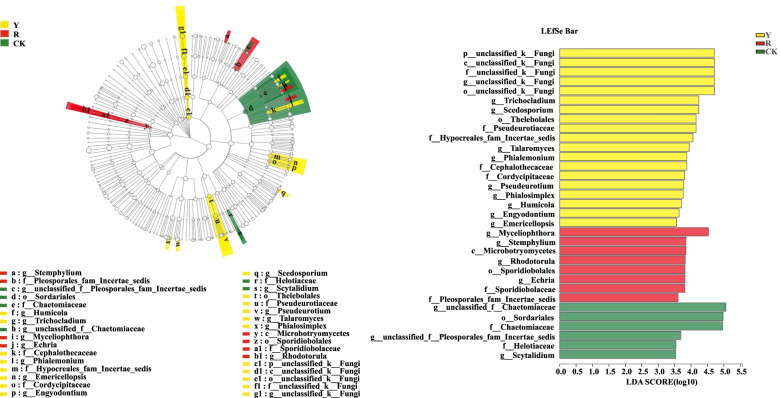
Lefse analysis of significant fungi among CK and the rhizospheres of yellow- (Y) and red-fruited tomatoes (R)

As shown in Fig. 9, the fungal compositions were significantly different in rhizospheres of yellow and red tomato varieties. Such as *Humicola* (genus), *Trichocladium* (genus), Cephalothecaceae (family), *Phialemonium* (genus), Hypocreales_fam_Incertae_sedis (from family to genus), Cordycipitaceae (from family to genus), *Scedosporium* (genus), Thelebolales (from order to family), *Pseudeurotium* (genus), *Talaromyces* (genus) and unclassified_k__Fungi (from phyla to genus) were enriched in rhizospheres of yellow fruit tomato varieties.

By contrast r, *Stemphylium* (genus), Pleosporales_fam_Incertae_sedis (family), *Microbotryomycetes* (genus), *Echria* (genus) and Sporidiobolales (from class to genus) were significantly enriched in rhizospheres of red tomato varieties. (Fig. 9).

To identify the co-occurrence patterns among species of abundant fungal genera, co-occurrence network analysis was performed.

**Fig. 10:**
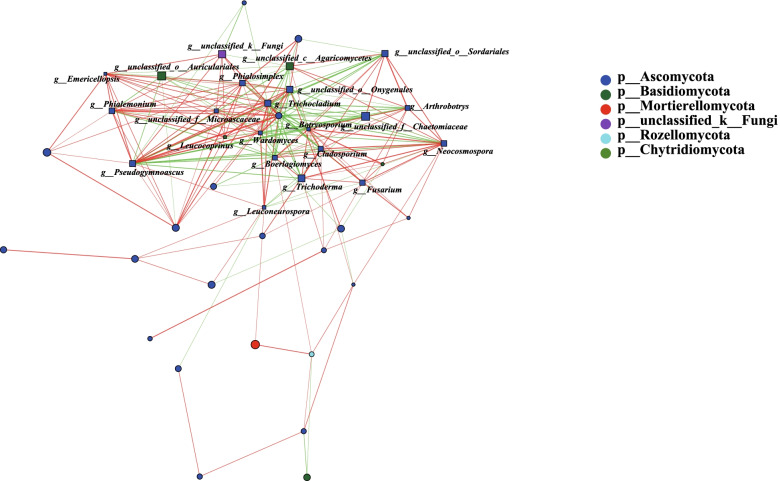
Co-occurrence network analysis of soil fungi in rhizospheres of different tomato varieties; The red line indicates a positive interaction, the green line indicates a negative interaction, and marked nodes represent significant differences, *p* < 0.05

As shown in Fig. 10, the resulting network showed the strongest correlation between the genus *Pseudogymnoascus* and the other fungal genera. In addition, *Trichocladium* had the second strongest correlation to other fungal genera, followed by *unclassified_o__Onygenales*, *Neocosmospora*, *Wardomyces*, *Boerlagiomyces*, *Botryosporium*, *Cladosporium*, *Trichoderma* and *Phialosimplex*. They are the top 10 soil fungi with the strongest correlation with other fungi. Most of them correlated positively. Interestingly, the phylum Ascomycota co-occurred with most of the other fungi from genera in the rhizospheres of tomato varieties with different fruit colors.

Genera exhibiting positive or negative correlations can describe the tendency of different fungal genera to co-occur or not in rhizospheres of yellow and red tomato varieties. Therefore, we can speculate that different fungal genera can be recruited by tomato varieties with different fruit colors. Two fungal genera exhibiting a significant positive correlation can grow better through metabolite exchanges, or two genera showing a significant negative correlation in abundance could directly interact through nutrient competition or differ in physiological requirements.

## Discussion

The contents of pigments in fruits of different tomato varieties leads to differences in color among their fruits. Studies have shown that the presence of β-carotene, anthocyanins and lycopene in different ratios leads to different colors on the fruit surface. Red tomato fruits have higher contents of lycopene and carotenoid than yellow tomato varieties. In contrast, no lycopene and a higher content of anthocyanin could be detected in yellow tomato varieties than in red tomato varieties [29].

Moreover, numerous studies have shown that plant hormones are closely associated with pigment formation. For example, auxin promotes lycopene accumulation [6]; Ethylene not only regulates carotenoid synthesis affecting fruit color changes [30] but can also increase lycopene content [31]. Moreover, abscisic acid not only regulates the carotenoid content [32] but also promotes lycopene synthesis for fruit reddening [33]. Furthermore, gibberellin acid inhibits carotenoid formation by inhibiting fruit reddening [34]. Likewise, the application of methyl jasmonate increased the *β*-carotene content and decreased the lycopene content in tomatoes, which affects fruit color changes [35].

On the other hand, abscisic acid has been confirmed to be significantly positively correlated with Actinobacteria and significantly negatively correlated with Proteobacteria [36]. Ethylene can be produced from *Nocardioides* [37], and auxin can be secreted from *Streptomyces* [38]. Gibberellin can be synthesized from *alpha-Proteobacteria* and *gamma-Proteobacteria* [39, 40], *Bradyrhizobium* [41], *Aspergillus* [42, 43] and *Penicillium* [44]. Moreover, pigments can also be produced by bacteria [45], e.g., *Bacillus* produces carotenoids [46], lycopene and β-carotene [47]; *Trichoderma* promoted increases in lycopene [48];

Actinobacteriota and Proteobacteria were found as the dominant soil bacterial phyla in rhizospheres of yellow and red tomato varieties. However, lower abundance of Proteobacteria and higher abundance of Actinobacteriota could be detected in the rhizospheres of yellow tomato varieties than those of red tomato varieties. This result suggested that the sources of abscisic acid in yellow tomato varieties could be speculated more abundant than that of red tomato varieties according to its correlation with Proteobacteria and Actinobacteria. Moreover, *Nocardioides*, which related to ethylene production,were detected as the unique dominant soil bacterial genera, *Streptomyces* and *Bacillus* was more abundant in the rhizospheres of red tomato varieties. Furthermore, *Trichoderma* was more abundant in the rhizospheres of red tomato varieties and showed prominent roles in cooccurrence network analysis. Based on these microbial functions, higher contents of auxin and ethylene in red-fruited tomato varieties than in yellow-fruited varieties could also assumed.

The abundance of Actinobacteriota in rhizosphere of yellow tomato varieties was also higher than that of red tomato varieties and showed a significant contribution to yellow tomato fruit color formation in the difference test. At the same time, the abundance of the dominant soil fungal genus *Aspergillus* was also higher in yellow than that in red tomato varieties. Moreover, *Penicillium* was the unique dominant soil fungal genera in yellow tomato varieties.

The results showed that Proteobacteria, *Aspergillus* and *Penicillium* may increase the endogenous gibberellin content of yellow-colored tomato varieties, and it can be inferred that the gibberellin content of yellow tomato varieties is higher than that of red tomato varieties. In addition, *Bradyrhizobium*, *Aspergillus*and and *Penicillium* are also considered as the gibberellines sources.

All of the above results confirmed that tomato varieties with different fruit colors recruited different functional soil microbes in the rhizosphere to produce plant hormones or were sources of different plant hormones related to fruit color formation.

## Conclusions

*Nocardioides*, *norank_f_norank_o_Vicinamibacterales*, *norank_f_norank_o_norank_c_KD4-96*, *norank_f_Birii41*, *norank_f_norank_o_S085* and *Bradyrhizobium* were the special soil dominant bacterial genera in the rhizospheres of red-fruited tomato varieties. In contrast, *unclassified_f__Micromonsporaceae*, *Acidipila*, *Roseisolibacter*, *Gaiella* and *norank_f_Xanthobacteraceae* were the unique soil dominant bacterial genera in the rhizospheres of yellow-fruited tomato varieties. In addition, *unclassified_o__Onygenales*, *Trichocladium*, *unclassified_c__Sordariomycetes*, *Pseudogymnoascus*, *Acremonium*, *Oidiodendron*, *Phialemonium*, *Penicillium*, *Phialosimplex* were the unique soil dominant fungal genera in rhizosphere of yellow tomato varieties. *Lecythophora*, *Derxomyces* and *unclassified_f_Pyronemataceae* were the unique soil dominant fungal genera in rhizospheres of red tomato varieties. Based on the functions of these special dominant soil bacteria and fungi in rhizospheres of yellow and red tomato varieties, it can be concluded that different soil microbes in rhizospheres are recruited by different tomato phenotypes related to tomato fruit color formation.

## Methods

### Field site description and experimental designs

**Fig. 11:**
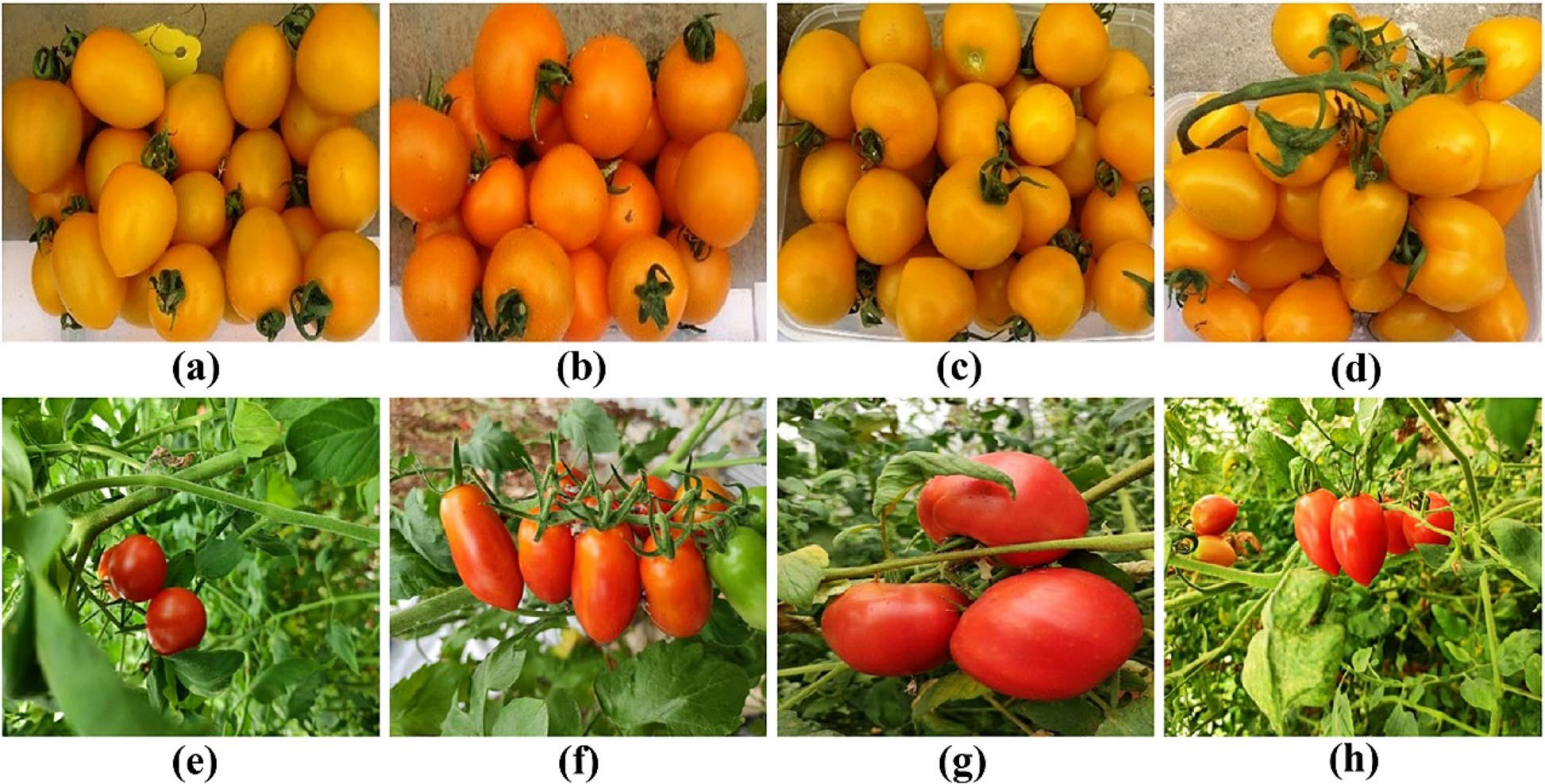
The appearance and morphological characteristics of the tomato varieties with different fruit colors

Two different colors tomato groups were used in this study, which d included four yellow-fruited tomato varieties (Jinniu 101 (a), Huang Xiaoya (b), Jimei No. 3 (c) and Milk Tomato (d)) and four red-fruited tomato varieties (Ally (e), Fengzhu (f), Taotaro (g) and Millenium (h). All above tomato varieties were purchased from Nong You Seedling Company (Fig. 11). All tomato varieties were identically treated and grew in the experimental station of the College of Agriculture, Guangxi University, Nanning (108°17′E and 22°51′N).

### Soil sampling and physicochemical properties

Rhizosphere soil samples were randomly collected by shaking method [49] during the fruit ripening stages. Briefly, three plants of each tomato variety were randomly selected, and then the whole plants including roots were dug out using a sterilized shovel. Meanwhile, soil samples from identical fields without any plant growth were also collected using as the CK.

The soil physical and chemical properties of the trial site were as follows: soil pH 5.68, organic matter content 8.92 g·kg^−1^, total nitrogen 0.55 g·kg^−1^, total phosphorus 0.67 g·kg^−1^, and total potassium 7.51 g·kg^−1^. The contents of alkaline dissolved nitrogen, available phosphorus and potassium were 15.27 mg·kg^−1^, 0.67 mg·kg^−1^, and 82.8 mg·kg^−1^, respectively.

### Test methods

#### Soil physicochemical properties

Soil pH value was determined with a pH meter (soil water ratio 1:2.5, w/ v); and the soil organic matter (SOM) content was determined by an external heating met hod using potassium dichromate [50]. Soil total nitrogen (TN) content was quantified by the Kjeldahl acid digestion method; soil total phosphorus (TP) content was quantified using the molybdate blue method after acid di gestion [51]. Soil total potassium (TK) was determined by alkali fusion flame spectrophotometry; soil available nitrogen (AN), phosphorus (AP) and potassium (AK) were subjected to the alkali diffusion method, double acid method and flame photometry, respectively [52].

##### Analysis of soil microbial diversity

Total DNA extraction, PCR amplification and sequence determination of the root samples were performed by Shanghai Majorbio Biopharm Technology Co., Ltd. High-throughput sequencing was performed using the MiSeq platform.

Total DNA extraction was performed according to the instructions of the FastDNA® Spin Kit for Soil (MP Biomedicals, U.S.), and DNA concentration and purity were measured using a NanoDrop 2000 spectrophotometer (Thermo Fisher Scientific, U.S.). PCR amplification was performed on an ABI GeneAmp® 9700 with the specific primers and sequencing types shown in Tables 3 and 4.

**Table 3:** Sequencing type and primer sequence

Sequencing type	Primer name	Primer sequence	Length	Sequencing platform
Bacterial 16SrRNA	338F	5′-ACTCCTACGGGAGGCAGCAG-3′	311 bp	MiseqPE300
	806R	5′-GGACTACHVGGGTWTCTAAT-3′

**Table 4:** Sequencing type and primer sequence

Sequencing type	Primer name	Primer sequence	Length	Sequencing platform
Fungal ITS	ITS1F	5′-CTTGGTCATTTAGAGGAAGTAA-3′	350	MiSeq PE300
	ITS2F	5′-GCTGCGTTCTTCATCGATGC-3′		

Sequencing was performed using Illumina's MiSeqPE250 platform (Shanghai Majorbio Biopharm Technology Co., Ltd.). PCR products from the same sample were purified using the AxyPrep DNA Gel Extraction Kit (Axygen Biosciences, Union City, CA, USA), mixed and detected by recovery using a 2% agarose gel. The recovered products were quantified using a Quantus™ Fluorometer (Promega, USA). Library construction was carried out using the NEXTFLEX® Rapid DNA-Seq Kit.

The PCR amplification of 16S rRNA gene was performed as follows: initial denaturation at 95 ℃ for 3 min, followed by 27 cycles of denaturing at 95 ℃ for 30 s, annealing at 55 ℃ for 30 s and extension at 72 ℃for 45 s, and single extension at 72 ℃ for 10 min, and end at 4 ℃. The PCR mixtures contain 5 × TransStart FastPfu buffer 4 μL, 2.5 mM dNTPs 2 μL, forward primer (5 μM) 0.8 μL, reverse primer (5 μM) 0.8 μL, TransStart FastPfu DNA Polymerase 0.4 μL, template DNA 10 ng, and finally ddH2O up to 20 μL. PCR reactions were performed in triplicate. The PCR product was extracted from 2% agarose gel and purified using the AxyPrep DNA Gel Extraction Kit (Axygen Biosciences, Union City, CA, USA) according to manufacturer’s instructions and quantified using Quantus™ Fluorometer (Promega, USA).

Illumina MiSeq sequencing: PCR products from the same sample were purified using the AxyPrep DNA Gel Extraction Kit (Axygen Biosciences, Union City, CA, USA), mixed and detected by recovery using a 2% agarose gel. The recovered products were quantified using a Quantus™ Fluorometer (Promega, USA). Library construction was carried out using the NEXTFLEX® Rapid DNA-Seq Kit.

The PCR amplification of 16S rRNA gene was performed as follows: initial denaturation at 95 ℃ for 3 min, followed by 27 cycles of denaturing at 95 ℃ for 30 s, annealing at 55 ℃ for 30 s and extension at 72 ℃for 45 s, and single extension at 72 ℃ for 10 min, and end at 4 ℃. The PCR mixtures contain 5 × *TransStart* FastPfu buffer 4 μL, 2.5 mM dNTPs 2 μL, forward primer (5 μM) 0.8 μL, reverse primer (5 μM) 0.8 μL, *TransStart* FastPfu DNA Polymerase 0.4 μL, template DNA 10 ng, and finally ddH_2_O up to 20 μL. PCR reactions were performed in triplicate. The PCR product was extracted from 2% agarose gel and purified using the AxyPrep DNA Gel Extraction Kit (Axygen Biosciences, Union City, CA, USA) according to manufacturer’s instructions and quantified using Quantus™ Fluorometer (Promega, USA).

Processing of sequencing data: The raw 16S rRNA gene sequencing reads were demultiplexed, quality-filtered by fastp version 0.20.0 [53] and merged by FLASH version 1.2.7 [54] with the following criteria: (i) the 300 bp reads were truncated at any site receiving an average quality score of < 20 over a 50 bp sliding window, and the truncated reads shorter than 50 bp were discarded, reads containing ambiguous characters were also discarded; (ii) only overlapping sequences longer than 10 bp were assembled according to their overlapped sequence. The maximum mismatch ratio of overlap region is 0.2. Reads that could not be assembled were discarded; (iii) Samples were distinguished according to the barcode and primers, and the sequence direction was adjusted, exact barcode matching, 2 nucleotide mismatch in primer matching [55].

Operational taxonomic units (OTUs) with 97% similarity cut off [56, 57] were clustered using UPARSE version 7.1, and chimeric sequences were identified and removed. The taxonomy of each OTU representative sequence was analyzed by RDP Classifier version 2.2 [58] against the 16S rRNA database using confidence threshold of 0.7.

Raw data were uploaded to the NCBI database for comparison. The data of the comparison database are as follows: bacterial for Silva (Release138, http://www.arb-silva.de); fungal for Unite (Release 8.0, http://unite.ut.ee/index.php).

### Statistical analyses

The data was statistically analyzed using Excel 2019 and Statistical Product and Service Solutions (SPSS) Statistics 21, And the R language (version 3.3.1) tool was used for Venn statistics and graphing. And R language (version 3.3.1) tool stats package and Python scipy package were used for difference test. Linear discriminant analysis (LDA) was performed using LEfSe (http://huttenhower.sph.harvard.edu/galaxy/root?tool_id=lefse_upload) on samples according to different grouping conditions based on taxonomic composition to identify clusters that had a significant differential impact on sample delineation. The results are shown as the means with their standard deviations (means ± SDs). Online data analysis was performed using the free online cloud platform (http://www.majorbio.com) of the Majorbio Bio-Pharm Technology Co. Ltd. (Shanghai, China).

## Data Availability

Raw data for bacterial and fungal bacterial sequence were deposited in the NCBI Sequence Read Archive (SRA) database under accession number PRJNA 859,555 and PRJNA 859,291, respectively.
